# Event-Related Desynchronization/Synchronization in Spinocerebellar Ataxia Type 3

**DOI:** 10.3389/fneur.2019.00822

**Published:** 2019-07-31

**Authors:** Yu Aoh, Han-Jun Hsiao, Ming-Kuei Lu, Antonella Macerollo, Hui-Chun Huang, Masashi Hamada, Chon-Haw Tsai, Jui-Cheng Chen

**Affiliations:** ^1^Neuroscience Laboratory, Department of Neurology, China Medical University Hospital, Taichung City, Taiwan; ^2^School of Medicine, College of Medicine, China Medical University, Taichung City, Taiwan; ^3^Sobell Department of Motor Neuroscience and Movement Disorders, UCL Institute of Neurology, London, United Kingdom; ^4^Department of Neurology, Graduate School of Medicine, The University of Tokyo, Tokyo, Japan; ^5^Department of Neurology, China Medical University Hsinchu Hospital, Hsinchu, Taiwan

**Keywords:** ERD, ERS, cerebellar ataxia, SCA3, motor control

## Abstract

**Introduction:** Spinocerebellar ataxia type 3 (SCA3) is an autosomal dominant, cerebellar degeneration predominant disease caused by excessive CAG repeats. We examined event-related dysynchronization/synchronization (ERD/ERS) in patients with SCA3.

**Methods:** We assessed ERD/ERS of self-paced voluntary hand movements in 15 patients with genetically proven SCA3 in comparison with healthy controls.

**Results:** In ERS, a significant interaction effect between group, frequency, and period (*F* = 1.591; *p* = 0.005; ρI = 0.86) was observed. The *post-hoc* two-tailed independent *t*-test showed significant differences in high beta and low beta ERS. By contrast, in ERD, no apparent differences were observed in the pattern of patients with SCA3 in comparison with healthy controls (*F* = 1.01; *p* = 0.442).

**Conclusion:** The study revealed a decreased ERS in patients with SCA3, especially at the frequency of 20–30 Hz. This study elucidates the significant role of cerebellum in motor control.

## Introduction

Spinocerebellar ataxia type 3 (SCA3, also known as Machado–Joseph disease) is an autosomal dominant, multisystem neurodegenerative disease caused by excessive CAG repeats (1). In addition to cerebellar ataxia, the clinical manifestations of SCA3 include dysarthria, progressive ophthalmoplegia, pyramidal signs, facial or lingual fasciculation, rigidity, dystonia, polyneuropathy, and lid retraction with bulging eyes. Postmortem studies have disclosed pathologic changes involving pons, spinocerebellar tract, Clarke column, anterior horn cells, substantia nigra, and the basal ganglia (2).

Electroencephalography is a non-invasive method that help us understand the pathogenesis of SCA3. A previous study on movement-related cortical potentials (MRCP) in patients with SCA3 revealed impaired cortical activation associated with affects the initiation and termination of voluntary movements (3). It suggested an impairment of the postsynaptic potentials on the pyramidal cell apical dendrites. The analysis of electroencephalogram (EEG) oscillation recorded during a motor task (self-paced movement) provided different information about the changes in cortical activity related to movement (4, 5), especially the neurons that involved in cortico-thalamic oscillations or other remote connectivities. In normal participants, event-related desynchronization (ERD) and event-related synchronization (ERS) are considered to indicate the activation and subsequent recovery of the motor cortex during planning, executing and completing a movement (4, 5). ERD and ERS are different responses of neuronal structures in the brain and are both time-locked to the event. In patients with neurodegenerative disorders, such as Parkinson's disease (PD) (6), multiple system atrophy (7), progressive myoclonic epilepsy (8), focal dystonia (9), and amyotrophic lateral sclerosis (10), the spatiotemporal pattern of ERD/ERS is altered. For example, in patients with writer's cramp, beta ERD in 20–30 Hz frequency bands significantly decreased. It may postulate the involvement of pyramidal system and basal ganglion in the genesis of ERD/ERS. However, research on ERD/ERS changes in patients with impaired cerebellar function is scant. Moreover, the mechanism through which cerebellar dysfunction affects the sensorimotor cortical activity is unclear. Therefore, we focused on the sensori-motor oscillatory changes in patients with SCA3 to study the information conveyed through voluntary movements. To the best of our knowledge, this is the first experiment aiming at understanding the ERD/ERS pattern changes in patients with SCA3.

Our study examined and elucidated the role of cerebellum and cortico-cerebellar pathway in motor control by observing the ERD/ERS pattern alteration in patients with clinical cerebellar syndromes caused by SCA3.

## Materials and Methods

Fifteen healthy human participants (mean age 43 ± 15.8 years, 7 women) and 15 patients with SCA3 (mean age 42 ± 12 years, 7 women) were enrolled in this study (Table 1). The patients included were genetically confirmed to have CAG tri-nucleotide repeats (mean repeats 69 ± 7). Disease duration was defined as the period from the first onset of symptoms to the time patients were included. The mean disease duration was 11.8 ± 11 years. All patients were evaluated by the same neurologist and were given a total score and sub-scores according to the clinical rating scale for cerebellar function (11). The mean score of the participants was 10 ± 7. All participants were right handed, and all gave their written informed consent. Some of the patients with SCA3 were previously evaluated in our MRCP study (3). The experiments conformed to the standards set by the Declaration of Helsinki and were approved by the China Medical University Hospital, Taiwan.

**Table 1:** Demographic data of the study patients with SCA3 and normal controls.

**Subject**	**Age**	**Sex**	**Disease duration (years)**	**CAG repeat**	**Clinical rating scale of Cerebellar function**	**Stance/Gait ataxia**	**Upper limbs ataxia (Right/Left)**	**Lower limbs ataxia (Right/Left)**	**Limbs hypotonia (Right/Left)**	**Postural tremor (Right/Left)**	**Dysarthria**	**Ocular movement**
1	56	M	7	*	7	1/3	0/1	0/0	0/0	0/1	0	1
2	21	F	7	80	1	0/0	0/0	0/0	0/0	0/0	0	1
3	44	M	30	82	10	0/1	1/1	1/1	0/0	1/1	1	1
4	54	M	40	61	23	4/3	3/3	1/1	0/0	1/0	1	5
5	53	F	1	66	17	1/1	1/1	1/1	1/1	1/1	1	6
6	59	F	17	80	16	3/3	3/3	3/3	1/1	0/0	0	1
7	35	F	0^#^	82	0	0/0	0/0	0/0	0/0	0/0	0	0
8	31	M	16	78	17	*	*	*	*	*	*	*
9	52	M	15	60	4	*	*	*	*	*	*	*
10	30	M	15	78	18	1/1	2/2	1/1	3/3	1/1	2	1
11	33	F	0^#^	70	4	0/0	0/1	0/0	0/0	0/0	0	3
12	33	M	8	76	7	1/2	0/1	0/0	0/0	1/1	1	1
13	48	F	3	71	6	1/1	1/1	0/0	0/0	0/0	0	2
14	31	F	4	76	11	1/2	1/1	1/1	0/1	1/1	1	0
15	52	M	14	71	17	2/2	1/2	1/1	1/1	1/1	1	2

^*^No data available.

# *Disease duration being zero means patient was clinically symptom-free*.

### EEG/EMG Recording and Preprocessing

During the self-paced palm-lifting task, EEG was recorded with 28 electrodes (Fp1, Fp2, F7, F3, Fz, F4, F8, FC3, FCz, FC4, T7, C3, Cz, C4, T8, CP3, CPz, CP4, P7, P3, Pz, P4, P8, O1, Oz, O2, EOG, and EMG) according to the international 10–20 system for EEG recording; bipolar surface EMG electrodes were placed on the right extensor carpi radialis at a spacing of 4 cm. Each participant was instructed to lift the right palm approximately every 6 s. After 1,000 s, ~60 wrist extension tasks each individual were recorded. We maintained the electrode impedance at <5 kΩ. While recording, the sampling rate was set at 1,000 Hz and online filtered with 0.05–70 Hz band-pass filter by using an amplifier (NeuroScan SynAmps, Neurosoft, Inc., Sterling, VA, USA). After recording, band-pass filter ranging from 1 to 50 Hz, resampling with 250 Hz, and average reference were applied during offline data analysis. The preprocessed data were subjected to an independent component analysis (ICA) decomposition by using EEGLAB. In addition, ICA components representing ocular and muscular artifacts were removed from the data.

### EMG

EMG was analyzed using EEGLAB 13_4_4b toolbox and MATLAB R2015a. For the chosen onset and offset markers, offline EMG signals were rectified and normalized using z-score. The threshold was defined as the absolute z-score value of >1. After identifying the threshold, the rectified raw EMG signals were epoched by onset markers between −50 and 1,000 ms and baseline corrected with the mean value between −3,000 and −2,000 ms. Peak amplitudes, peak latencies, and duration of EMG were collected within the time window.

### Time-Frequency Power Analysis

Computed event-related spectral perturbations (ERSP) were analyzed through Fast-fourier transformation using a three-cycle wavelet Hanning-tapered window for providing a continuous measure of the amplitude of a frequency component (12). ERSP of the wavelet-transformed epochs were computed for each stimulus requirement at every time point and wavelet frequency to yield time–frequency maps. Latency time relative to the time-locking event and amplification or attenuation at a given frequency were then illustrated using a color for each image pixel. After ERSP analysis, the absolute power was computed using the equation ersp_abs = 10$.^{\wedge }\left(\frac{ersp\left\{1\right\}}{10}\right)$, where ersp is the outcome log spectral differs from baseline which obtain from ERSP analysis.

### ERD/ERS Analysis

ERD/ERS calculation was performed according to the method of Pfurtscheller and Aranibar (13). The ERD/ERS value was calculated by averaging the absolute power which obtained from ERSP analysis according to different range of frequency bands. The outcome ERD/ERS plots were drew from −3 to 4.5 s. The baseline period was from −3 to −2 s. The frequency bands of interest were alpha (8–12 Hz), low beta (13–20), and high beta (20–30) bands. The EEG data were then smoothed at every 150 ms in one value.

### Statistical Analysis

Multivariate repeated measures analyses of variance was performed using IBM SPSS (Statistical 22, IBM Corp., USA software) to assess movement-related power change in C3 electrode in SCA 3. The within-subject factors: frequency [alpha (8–12 Hz), low beta (13–20), and high beta (20–30)] and a given period (ERD: −3 to 1 s, ERS: 0.5 to 4.5) and between-subject factor: groups (2 levels: SCA 3 and control) were performed. This resulted in a 3-way repeated GLM (frequency^*^period^*^group) in ERD or ERS analysis separately.

Having identified the interaction effect of frequency^*^period^*^Group, we then assessed in a second step period and group effects in a two-way repeated measures GLM (Period^*^Group) in each frequency band.

Finally, follow-up pairwise comparisons were run to assess the effect within levels of the group factor (SCA 3 and control). Only effects with effect sizes >0.35 (based on the intraclass correlation coefficient: ICC) were considered for follow-up analyses to avoid reporting non-essential effects (14). Greenhouse-Geisser-corrected results were reported when assumptions of sphericity were not met and Bonferroni correction was used for pairwise comparisons.

Pearson's correlation coefficient was used to investigate the correlation between clinical rating scale for cerebellar function and peak ERD/ERS in each band. We further analyzed individual subscales with the significant band.

## Results

The participants were 15 controls and 15 patients with SCA3 (Table 1). No difficulty was experienced by the patients and controls in completing the tasks. No significant age- or sex-related differences were observed (*p* = 0.83; *p* = 1).

**Figure 1:**
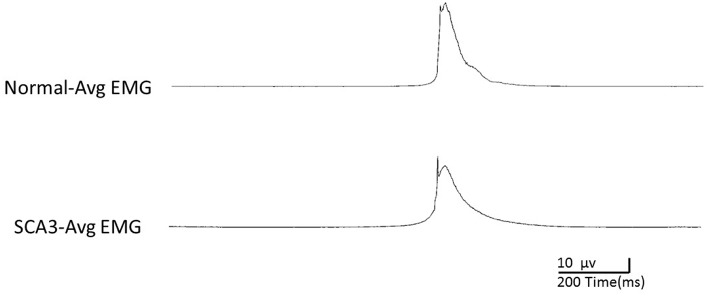
Illustration of processed averaged EMG of Normal and SCA3 during the EEG recording.

Table 2 shows the EMG peak latency, mean duration, and amplitude in the two groups. The average rectified EMG duration and peak amplitude between the two groups were not statistically different (*p* = 0.27; *p* = 0.87). In patients with SCA3, peak latency was slightly delayed (mean difference = 45.9 ms, *p* = 0.03; Figure 1).

**Table 2:** The EMG characters of SCA3 & controls.

**Duration & Latency Average**			
**Character**	**Duration (ms)**	**Latency (ms)**	**Amplitude (μV)**
Normal	265.4 ± 109.0	107.5 ± 37.6	695.4 ± 434.3
SCA3	311.9 ± 115.9	153.4 ± 70.3	672.2 ± 300.4
P value	0.27	0.03^*^	0.87

* *P < 0.05*.

**Figure 2:**
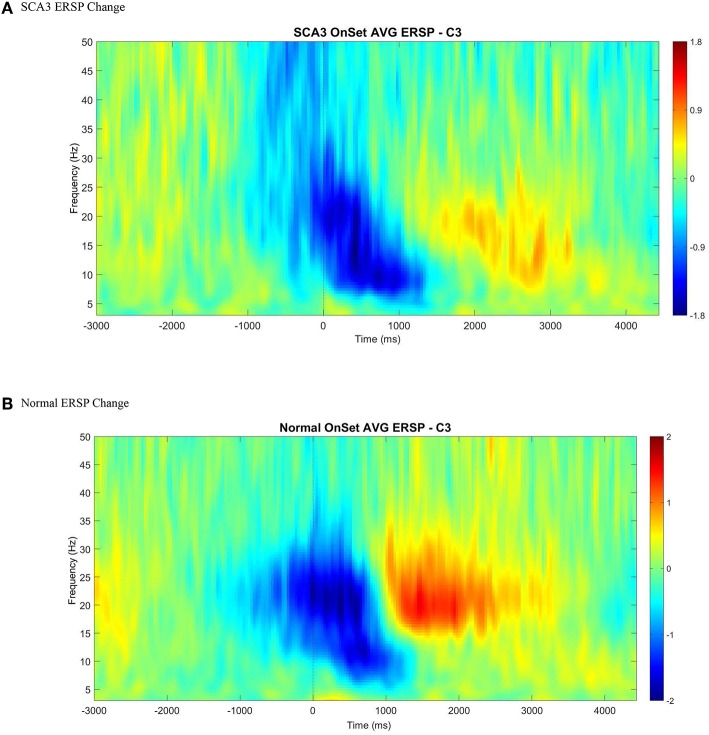
An illustration of averaged event-related spectral perturbation at movement onset (0 ms) across all control **(B)** and SCA3 **(A)**. A clear ERD (color blue) followed by ERS (color red) was presented in both control and SCA3 groups between alpha and beta range at C3 electrode when epoched between −3 and 4.5 s with baseline corrected by −3 to −2 s.

Among the 15 patients with SCA3, similar to the healthy controls, we found a clearly detectable temporospatial pattern of movement-related cortical activities, indicating ERD and ERS around the movement onset (Figures 2A,B).

### ERD Analysis

**Figure 3:**
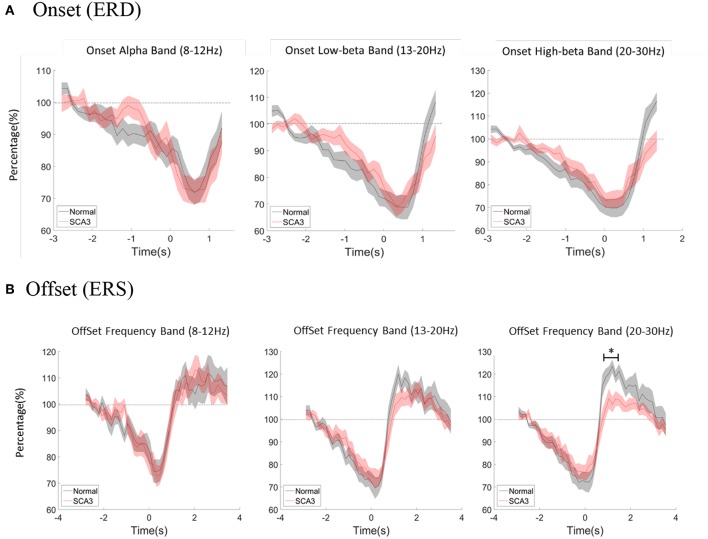
**(A)** Averaged power change at movement onset between alpha, low beta, and high beta bands both in normal controls and SCA3 patients. **(B)** Averaged power change at movement offset between alpha, low beta, and high beta bands in both control and SCA3 groups. *post-hoc* independent *t*-test showed significant difference in high beta ERS (marked as ^*^).

Multivariate repeated measures analyses of variance was used to investigate the relative power difference of ERD/ERS. In ERD (Figure 3A), 3-way repeated GLM (frequency^*^period^*^group) were performed. Neither the main effect of group nor interaction effect of Group^*^Frequency was observed, which indicates that the frequency distribution of ERD was similar in both groups (*F* = 0.466; *p* = 0.630; Table 3). Furthermore, no interaction effect of Group^*^Period was observed, which indicates that the time course of ERD was similar in both groups (*F* = 1.318; *p* = 0.134). In addition, Group^*^Frequency^*^Period did not have a significant interaction effect (*F* = 1.017; *p* = 0.442).

**Table 3:** Three-way repeated measure analysis of variance (frequency^*^period^*^group) results in ERD/ERS^**^ between Normal and SCA3.

**On-Set ERD**	***P*-value**	***F***	**Off-Set ERS**	***P*-value**	***F***
Group	0.417	*F*_(1, 28)_ = 0.680	Group	0.365	*F*_(1, 28)_ = 0.849
Group^*^ Frequency	0.630	*F*_(2, 56)_ = 0.466	Group^*^ Frequency	0.206	*F*_(2, 56)_ = 1.623
Group^*^ Period	0.134	*F*_(49, 1372)_ = 1.318	Group^*^ Period	0.923	*F*_(49, 1372)_ = 0.633
Group^*^ Frequency^*^ Period	0.442	*F*_(98, 2744)_ = 1.017	Group^*^ Frequency^*^ Period	0.005^*^	*F*_(98, 2744)_ = 1.591

* *P < 0.05*.

** *ERD period start from −1 to 1 s, ERS period start from 0.5 to 4.5 s*.

### ERS Analysis

With the similar statistics, in ERS (Figure 3B), the main effect of group and interaction effect of Group^*^Frequency was not observed, which indicates that the frequency distribution of ERS was similar in both groups (*F* = 1.623; *p* = 0.206). Furthermore, no interaction effect of Group^*^Period was observed, which indicates that the time course of ERD was similar in both groups (*F* = 0.633; *p* = 0.923). However, a significant interaction effect of Group^*^Frequency^*^Period (*F* = 1.591; *p* = 0.005; ICC = 0.86) in Table 3.

In a second step, we further tested the Group^*^Period interaction effect in each frequency band. Only interaction effect in high beta frequency was significant (alpha *F* = 0.437; *p* = 0.994; low beta *F* = 1.446; *p* = 0.71; high beta *F* = 2.191; *p* = 0.001; ICC = 0.36).

The *post-hoc* pairwise comparison was focused on high beta ERS (0.5–4.5s) and showed significant differences as described in Table 4. Each period represented 150 ms after movement offset. The differences of ERS were noted between P3 ~ P11 which is around 1 to 2 s after movement offset.

**Table 4:** The *post-hoc* two-tailed independent *t*-tests of ERS (0.5–4.5 s) between SCA3 and control in high beta bands.

**Period**	**P1**	**P2**	**P3**	**P4**	**P5**	**P6**	**P7**	**P8**	**P9**	**P10**	**P11**	**P12**	**P13**	**P14**	**P15**	**P16**	**P17**	**P18**	**P19**	**P20**	**P21**	**P22**	**P23**	**P24**	**P25**	**P26**	**P27**
**High Beta**	0.803	0.642	0.035^*^	0.016^*^	0.066^**^	0.002^*^	0.024^*^	0.002^*^	0.007^*^	0.056^**^	0.048^*^	0.106	0.191	0.269	0.199	0.391	0.284	0.217	0.729	0.394	0.646	0.701	0.533	0.848	0.531	0.833	0.895

*Each period represented 150 ms after movement offset. The differences of ERS were noted between P3 and P11 which is around 1–2 s after movement offset*.

* p < 0.05;

** *P < 0.1. Bold values indicates P < 0.05*.

The correlation between the clinical rating scale for cerebellar function and high beta ERS was *R* = 0.44, *p* = 0.10. We also assessed the correlation in clinical rating subscale for high beta ERS. Table 5 shows the clinical rating scale for cerebellar function and the individual subscale as well as the correlation of high beta ERS and *p*-value. No significant correlation was noted in the clinical score with ERS (Table 5).

**Table 5:** Pearson's correlation of Clinical Rating Scale for Cerebellar Function with high beta ERS.

**High beta ERS**	**Clinical rating scale for cerebellar function**	**Standing balance**	**Gait ataxia**	**Upper limb ataxia(R)**	**Upper limbs ataxia L**	**Lower limb ataxia(R)**	**Lower limbs ataxia L**
Correlation	0.440	0.481	0.540	0.525	0.442	0.544	0.544
*P*-value	0.101	0.096	0.057	0.066	0.131	0.055	0.055
**High beta ERS**		**Limbs hypotonia(R)**	**Limbs hypotonia (L)**	**Postural tremor(R)**	**Postural tremor(L)**	**Dysarthria**	**Ocular movement**
Correlation		−0.067	0.154	0.363	0.152	0.224	0.203
*P*-value		0.828	0.615	0.223	0.62	0.463	0.506

## Discussion

We demonstrated a prominent reduction of beta ERS, especially at a frequency of 20–30 Hz, in patients with SCA3. However, no apparent differences were observed in the ERD of the patients compared with the healthy controls. To our knowledge, this is the first report on the pattern changes of ERD and ERS in patients with SCA3.

Brain oscillations are produced by neuronal networks. The activation and deactivation of these networks can result in oscillatory changes in the synchrony of cell populations due to externally or internally paced events and can lead to characteristic EEG patterns. Two such pattern types were observed: ERD and ERS (15). ERD and ERS indicate the activation and subsequent recovery, respectively, of the motor cortex in normal people while planning, executing, and completing a movement (4, 5).

Generally, ERD can be interpreted as an electrophysiological correlate of an increased cortical excitability or an activated cortical area (16). It is also a reliable marker of increased neuronal excitability. Various studies have discussed the pathophysiologic role of ERD in several neurologic diseases, such as PD (6) and focal dystonia (9). This role may be related to pathophysiological changes occurring in the basal ganglia, inducing impaired dopaminergic modulation of cortical activation. In patients with writer's cramp, beta ERD in 20–30 Hz frequency bands significantly decreased. The underlying pathophysiology might be the failure of motor cortical activation secondary to aberrant basal ganglia inputs (9). In our patients, ERD was comparable with that in healthy controls. Compared with the aforementioned basal ganglia disorders, cortical activation represented by ERD is relatively preserved in patients with SCA3.

ERS indicates the local inhibition of motor cortex below the resting baseline (17) or excitation removal back to the baseline level (18) or both (19). ERS in the alpha and/ lower beta bands represents cortical deactivated state and decreased information processing (20). Thus, ERS could be regarded the inhibition or termination signal of motor order. Moreover, ERS reinforces existing motor states and steady motor output (21). Because beta oscillations were evident across the cortical-basal ganglia sensorimotor network, beta ERS was abnormal in various neurologic diseases. In patients with Alzheimer disease (22) and stroke (23), the spatiotemporal pattern of beta ERS is definitely altered due to cortical involvement. Furthermore, patients with PD (24), paroxysmal kinesigenic dyskinesia (25), myoclonus-dystonia (26), writer's cramp27 and Unverricht-Lundborg disease (8) have decreased beta ERS amplitude. Moreover, ERS between 10 and 13 Hz may represent a deactivated cortical area or inhibited cortical network. Meanwhile, induced beta oscillation(13–35 Hz) were found in sensorimotor areas after voluntary movement and after somatosensory stimulation, which might be interpreted as a state of “inhibition” of neural circuitry in the primary motor cortex (16). In patients with impaired basal ganglia-cortical motor loop, net dopamine levels modulate their beta oscillatory activity (27). In addition to the association between ERS and the defective basal ganglia-cortex motor circuit resulting in impaired cortical motor inhibition, researchers also found an association between ERS and the processing of sensory afference. In patients with PD, beta ERS was significantly lowered in the patient group after manipulation of sensory input, voluntary movement and passive movement. Therefore, the decreased ERS might indicate a deficient sensorimotor integration in patients with PD (28).

Moreover, a recent study suggested that ERS is related to processes exercising Bayesian inference in sensorimotor integration in determining revision or maintenance of the existing motor plan. Furthermore, the amplitude of ERS indexes the confidence attributed to feedforward estimations relative to sensory feedback. In healthy participants, higher ERS amplitude represents higher confidence in maintaining stable motor output, whereas lower ERS amplitude indicates lower confidence in feedforward estimations and the need for adaptive changes driven by sensory feedback (29).

For optimal movement control, the coordination of inputs from peripheral sensation, cerebellum, basal ganglia, and sensorimotor cortical integration is mandatory. Particularly, the cerebellum plays a significant role in high-order motor control. Studies have revealed how cerebellar pathways integrate with the sensory, pyramidal, and extra-pyramidal system. An extensive cerebellar–pons–cortical pathway was discovered in mice, which contributed to the fine motor control of the vibrissa system in order to adjust to the surrounding environment. A zone in the cerebellar cortex was also identified where primary sensory and motor cortical inputs converge at the cellular level, providing the anatomical evidence of the existence of sensorimotor cortico-cerebellar loops and showing its importance in the fine control of voluntary movements (30). With the input of both sensory feedback and motor command, the cerebellum was also considered to be involved in the estimation of future state in a dynamic system (31).

Although the cerebellum has a clearly indispensable role in voluntary motor control, it was not previously investigated with respect to change in ERS pattern. Our study showed decreased high beta ERS in patients with SCA3 mainly manifesting with cerebellar ataxia, indicating a defective inhibition of motor order, failed reinforcement of current motor state, and impaired sensorimotor integration causing low confidence in feedforward estimation to maintain steady motor output, which was most likely being related to damaged cerebellum and cerebello-cotrical pathways. A prior study of MRCP in patients with SCA3 also provided evidence of impaired termination of a voluntary movement (3). Clinically, this could possibly explain the imprecise termination of movement in patient with SCA3, manifesting with overshooting, dysdiadochokinesia, and failure in performing the finger-chasing test. The present study revealed the ineligible influence of cerebellum and its projection to the cortex and motor neural network by observing the significant reduction of beta ERS. Sensori-motor integration abnormality may play a role in this finding.

However, the clinical scores did not show an obvious correlation with patient's ERS power. This result may be due to the small sample size or the related small scale of each individual score in the clinical cerebellar score.

The decreased beta ERS in patients with PD has been restored through deep brain stimulation or administration of levodopa, which, from a neurochemical point of view, indicates that neural circuits interfering with beta ERS might be partially dopaminergic (7, 27, 32). As explained earlier, beta ERS could be an indicator of confidence in current motor state and maintenance of steady motor output. However, uncertainty had been discovered to be related to acetylcholine and norepinephrine signaling (33). Therefore, the elements comprising beta ERS involved numerous neural networks and neuromodulators.

The present study has some limitations. First, the statistical significance notwithstanding, the sample size of the study is relatively small. A larger sample size is needed for a more comprehensive evaluation of changes in movement-related neural activities in patients with SCA3. Second, the clinical rating scale for cerebellar function (11) included all essential elements of the clinical picture of a cerebellar patient. However, it was less frequently used than the scale for the assessment and rating of ataxia score (SARA) (34) as the total score of clinical rating scale for cerebellar function placed too much emphasis on oculomotor manifestations. Last, SCA3 *per se* is a neurodegenerative disease with multisystem involvement, including the basal ganglia (35). Although our patients mainly manifest with cerebellar ataxia and the clinical signs indicating extrapyramidal system impairment were not prominent, the interference of beta ERS by the basal ganglia–cortical pathways could not be eliminated completely. Additional investigations of ERD/ERS are required in other neurologic diseases, such as SCA6, that involve solely the cerebellar system.

In this study, we found a decreased ERS in patients with SCA3, which represents the involvement of cerebellar system in motor termination. The reflection of impaired motor deactivation, sensorimotor integration, or sensory feedforward estimation may be related to the underlying mechanism. Hitherto, there are no effective treatments for patients with hereditary spinocerebellar ataxias. However, a clearer understanding of the pathophysiologic pathways of SCA3 could possibly prompt novel understanding for this group of patients in the future.

## Ethics Statement

This study was carried out in accordance with the recommendations of Human Subjects Research Act, Ministry of Health. The protocol was approved by the Ethic committee of China medical university hospital. All subjects gave written informed consent in accordance with the Declaration of Helsinki.

## Author Contributions

C-HT and M-KL provided patients with confirmed diagnosis. YA contributed to clinical scoring of patients. YA and H-JH wrote the manuscript with support from J-CC. H-JH performed the analytic calculations. C-HT, M-KL, AM, MH, and H-CH provided critical feedback and helped shape the research, analysis, and manuscript. All authors discussed the results and contributed to the final manuscript.

### Conflict of Interest Statement

The authors declare that the research was conducted in the absence of any commercial or financial relationships that could be construed as a potential conflict of interest.
